# Identification and Validation of Potentially Clinically Relevant CpG Regions within the Class 2 Tumor Suppressor Gene *SFRP1* in Pancreatic Cancer

**DOI:** 10.3390/cancers15030683

**Published:** 2023-01-22

**Authors:** Maximilian Hauschulz, Sophia Villwock, Jennifer Kosinski, Florian Steib, Lara Rosaline Heij, Jan Bednarsch, Ruth Knüchel-Clarke, Edgar Dahl

**Affiliations:** 1 Institute of Pathology, Medical Faculty, RWTH Aachen University, D-52074 Aachen, Germany; 2 Center for Integrated Oncology Aachen Bonn Cologne Duesseldorf (CIO ABCD), D-52074 Aachen, Germany; 3 Department of Surgery and Transplantation, Medical Faculty, RWTH Aachen University, D-52074 Aachen, Germany; 4 RWTH centralized Biomaterial Bank (RWTH cBMB), Medical Faculty, RWTH Aachen University, D-52074 Aachen, Germany

**Keywords:** pancreatic cancer, *SFRP1*, *SFRP1* promoter hypermethylation, class 2 tumor suppressor genes, pyrosequencing, chemotherapy, FFPE

## Abstract

**Simple Summary:**

Pancreatic cancer is one of the deadliest cancer entities with five-year survival rates of less than 11%. Besides standardly used surgical therapy, available chemotherapies are increasingly used to prolong overall survival. Promoter hypermethylation of the tumor suppressor gene *Secreted frizzled-related protein 1* (*SFRP1*) seems to be correlated with poor response to gemcitabine treatment in stage IV pancreatic cancer. The aim of this study was to find and characterize key CpG sites in the promoter region of the *SFRP1* gene. We identified a core CpG island whose DNA methylation may have a decisive influence on *SFRP1* expression loss and unfavorable overall survival. Its specific analysis may predict response of stage IV tumors to chemotherapy in the future.

**Abstract:**

In pancreatic cancer treatment, tumor stage-dependent chemotherapies are used to prolong overall survival. By measuring DNA promoter hypermethylation in the plasma of patients with stage IV pancreatic cancer, it was recently shown that promoter DNA methylation of the tumor suppressor gene *SFRP1* has a high value for predicting failure of drug treatment with gemcitabine. In this study, we therefore aimed to identify as precisely as possible the region in the *SFRP1* promoter that is frequently hypermethylated in pancreatic cancer tissue. First, we used the TCGA data set to define CpG-rich regions flanking the *SFRP1* transcription start site that were significantly more methylated in pancreatic cancer compared to normal pancreatic acinar tissue. A core CpG island was identified that exhibited abundant tumor DNA methylation and anti-correlation of *SFRP1* mRNA expression. To validate our in silico results, we performed bisulfide conversion followed by DNA pyrosequencing of 28 matched formalin-fixed, paraffin-embedded (FFPE) pancreatic cancer cases and six pancreatic cancer cell lines. A defined block of seven CpG sites within the core CpG island was identified, which confirmed our in silico results by showing significantly higher *SFRP1* methylation in pancreatic cancer specimens than in normal pancreatic tissue. By selecting this core CpG island, we were able to determine a median overall survival benefit for the low *SFRP1* methylation group compared to the high *SFRP1* methylation group (702 versus 517 days, *p* = 0.01) in the TCGA pancreatic cancer cohort. We propose a compact pyrosequencing assay that can be used in the future to further investigate the prognostic value of *SFRP1* promoter hypermethylation in predicting pancreatic cancer chemoresistance. Therefore, instead of DNA analysis from blood (liquid biopsy), DNA easily extractable from cancer tissue blocks (FFPE material) could be used.

## 1. Introduction

Pancreatic cancer represents one of the deadliest cancer entities with a worldwide annual burden in 2018 of estimated 458,918 new cases and around 432,000 cancer deaths [1]. Even though most newly diagnosed pancreatic cancer cases appear in developed countries [1], five-year survival rates have not improved considerably in the last decade and do not exceed 11% [2]. Observing the increasing therapeutic success in other tumor entities, it has therefore been predicted that pancreatic cancer will become the second leading cause of cancer related death by 2030, right behind bronchial and lung carcinomas [3]. One main cause for these poor treatment prospects is the late timepoint of diagnosis. Pancreatic cancer, especially at early stages, is a clinical silent disease with mostly nonspecific symptoms such as weight loss, nausea and weakness [4]. Therefore, only around 13% of the cases are diagnosed at localized stage, while around 77% are diagnosed at advanced stages (locally advanced or metastatic) [2]. Only at a regional state are the patients eligible for surgical resection, which is the only potential curative therapy by now [5]. Besides improving surgical techniques, complications during or after intervention, e.g., post-operative pancreatic fistulas, are still common and result in notable perioperative morbidity and mortality [6,7]. Although available chemotherapy regimens such as gemcitabine/paclitaxel or mFOLFIRINOX can increase overall survival [8], not all patients are suitable for these therapies, so there is plenty of room for more advanced drug-based therapy options such as targeted therapy approaches. Frequent and well-known genetic alterations in pancreatic cancer include those of *KRAS*, *CDK2A*, *TP53* and *SMAD4* genes [9], which are possible targets for small molecule approaches in multiples studies but unfortunately not yet clinically established therapy options [10,11].

One cellular signaling pathway that seems to be involved in pancreatic cancer development and maintenance of cancer stem cells is the canonical Wnt pathway [12,13], which regulates nuclear β-catenin concentration by degradation via the so-called destruction complex built from adenomatosis polyposis coli (APC), glycogen synthase kinase 3 beta (GSK-3β) and axin proteins. If Wnt ligands bind to the transmembrane receptor frizzled and its coreceptor low-density lipoprotein receptor-related protein 5 and 6 (LRP5/6), intracellular located dishevelled (Dsh) is activated, resulting in inhibition of the destruction complex. Therefore β-catenin is no longer degraded and can translocate into the nucleus [14,15]. The pathological effect of this Wnt pathway activation has been intensively studied in colorectal carcinoma, where APC is typically mutated, and, with that, elevated β-catenin levels lead to cancer development [16]. In pancreatic cancer, direct Wnt pathway activation by upregulation of Wnt family member 2 (WNT2) and Wnt family member 7A (WNT7A) has frequently been observed, resulting in poor clinical outcomes [17,18]. Moreover, indirect activation of the WNT pathway seems to be a common mechanism. For example, a crosstalk has been reported between elevated classic WNT/β-catenin activity in pancreatic cancer and abundantly expressed hypoxia-inducible factor 2α (HIF2α), together promoting tumor proliferation, invasion and stemness [19].

In contrast to ligand-dependent Wnt pathway activation, loss of well-known negative regulators seems to occur in pancreatic cancer as well. Secreted frizzled-related protein 1 (SFRP1) is an important negative regulator of the Wnt pathway, which can suppress pathway activation by binding of Wnt ligands [20]. In human cancers, the *SFRP1* gene belongs to the group of class 2 tumor suppressor genes (C2TSG) [21,22] that are frequently silenced by promoter DNA hypermethylation [23,24,25,26]. A recent study by Stubbe et al. revealed that *SFRP1* promoter methylation, detected by methylation-specific polymerase chain reaction (MSP) in blood serum, seems to be an independent predictive marker for gemcitabine treatment response in stage IV pancreatic ductal adenocarcinoma patients [27]. 

Our aim of this study was to identify those regions of the *SFRP1* promoter DNA sequence as precisely as possible, which are frequently hypermethylated in pancreatic cancer and involved in downregulation of *SFRP1* expression. Therefore, we first identified promising CpG site sequences in silico and verified them by pyrosequencing after DNA bisulfite conversion. To our best knowledge, studies on *SFRP1* promoter methylation in pancreatic cancer tissue were performed, so far, mostly by MSP, which is not a quantitative technique and, thus, may not be optimal to develop clinical assays. Additionally, we focused on detecting DNA methylation directly on cancerous tissue samples. For that purpose, DNA was extracted from formalin-fixed paraffin-embedded pancreatic cancer and normal acinar tissues, as well as from pancreatic cancer cell lines. 

## 2. Material and Methods

### 2.1. In Silico Analysis

For in silico analysis, the data of “TCGA Pancreatic Cancer (PAAD)” available via the software Xena Browser (University of California, Santa Cruz, CA, USA) were used. Only data for “Solid Tissue Normal” and “Primary Tumor” were analyzed. The focus was set on the Infinium HumanMethylation450 BeadChip data, the RNASeqV2 data and the overall survival data. The genomic DNA sequence for *SRFP1* was obtained from the UCSC Genome Browser Human GRCh 37/hg19 data set (University of California, Santa Cruz, CA, USA). For the identification of CpG islands prone to hypermethylation in the *SFRP1* sequence, MethPrimer software (Peking Union Medical College Hospital, Beijing, China) was used. To do this, detection criteria were set as default with CpG island size > 100 bp, CG percentage > 50% and CpG ratio > 60%.

### 2.2. FFPE Patient Collective and DNA Isolation

FFPE tissue samples of 28 pancreatic ductal adenocarcinoma patients with corresponding adherent normal acinar pancreas tissues and seven corresponding metastases, including one liver metastasis and six lymph node metastases, were analyzed. The clinico-pathological characteristics of the FFPE patient collective are shown in Supplementary Table S2. All patients underwent surgery at the Department of General, Visceral and Transplant Surgery of the University Hospital RWTH Aachen in the years from 2012 and 2015. The study was evaluated and approved by the Institutional Review Board of the Medical Faculty of the RWTH Aachen University (EK 100/21) and conducted in accordance with the principles of the Declaration of Helsinki and Good Clinical Practice guidelines (ICH-GCP). For every tissue sample, 2 µm sections were prepared and stained with hematoxylin and eosin (HE). For DNA analysis, an experienced pathologist examined relevant areas of the tissues, i.e., for cancer tissue areas with high tumor to connective tissue ratio and for normal pancreatic acinar and metastasis tissue areas with only cells of interest. After deparaffination (xylol, ethanol), the relevant tissue regions were macrodissected from 10 µm sections. For DNA isolation out of the collected tissue, the QIAamp DNA Mini Kit (QIAGEN, Hilden, Germany) was used. In addition to DNA isolation from FFPE tissue samples, DNA from six pancreatic cancer cell lines was isolated, whereby isolation was performed according to manufacturer’s instructions. As recommended, all samples were treated with ribonuclease A from bovine pancreas (Sigma-Aldrich, Deisenhofen, Germany). The DNA concentration of all samples was measured using the NanoDrop ND-1000 Spectrophometer (VWR, Radnor, USA), and samples were stored at −20 °C.

### 2.3. Cell Culture

The pancreatic cancer cell lines PANC-1, MIA PaCa-2, BxPc-3 and AsPC-3 were provided by the Department of General, Visceral and Transplant Surgery, University Hospital RWTH Aachen. PSN-1 and DAN-G cell lines were provided by the Institute of Pathology, University Hospital HHU Düsseldorf. All human cell lines were authenticated by single nucleotide polymorphism (SNP) profiling provided by Multiplexion GmbH (Immenstadt, Germany). PANC-1 and MIA PaCa-2 cells were cultivated in Gibco^TM^ DMEM high glucose medium and PSN-1, BxPc-3, AsPC-1 and DAN-G cells in Gibco^TM^ RPMI 1640 medium, each supplemented with 10% fetal bovine serum and 1% L-glutamine-penicillin-streptomycin. For BxPc-3 and AsPC-1, the medium was additionally supplemented with 1% sodium pyruvate. All cell lines were cultivated under standard conditions with 37°C, 5% CO_2_, 20% O_2_ and 95% humidity; the medium was changed twice a week, and cells were divided at 80% confluency. Additionally, the cells were regularly tested for mycoplasma contamination. 

### 2.4. RNA Isolation and RT-PCR

Cell line RNA samples were isolated using the NucleoSpin^TM^ RNA Plus Kit (Machery-Nagel, Düren, Germany) according to the manufacturer’s instructions. RNA concentrations were measured with the NanoDrop ND-1000 Spectrophometer (VWR, Radnor, PA, USA). For long term storage, samples were kept at −80 °C.

The Reverse Transcription System (Promega, Madison, MI, USA) was used for reverse transcription of RNA samples according to the manufacturer’s instructions. Real-time polymerase chain reaction (RT-PCR) was performed on the CFX96 Touch Real-Time PCR System (Bio-Rad Laboratories, Munich, Germany) with the primers for the gene of interest *SFRP1* and the reference gene *ACTB* (Metabion International AG, Planegg/Steinkirchen, Germany, Supplementary Table S1). cDNA was amplified using the iTaq^TM^ Universal SYBR Green Supermix (Bio-Rad Laboratories, Munich, Germany) according to the manufacturer’s instructions. Briefly, the performed RT-PCR program was set as 95 °C for 30 s, followed by 40 cycles of 95 °C for 5 s and 60 °C for 30 s, with following melting curve analysis starting from 65 °C to 95 °C with 0.5 °C temperature increase per 5 s. For analysis, non-detectable expression levels were set as Ct = 40.

### 2.5. Pyrosequencing

In preparation for pyrosequencing, 500 ng DNA were bisulfide-converted for 16 h using the EZ DNA Methylation-Lightning Kit (Zymo Research, Bad Homburg, Germany) according to the manufacturer’s instructions. Pyrosequencing was performed on the PyroMark Q96 ID sequencer (Qiagen, Hilden, Germany) using the components PyroMark PCR Kit, PyroMark Gold Q96 Reagents, PyroMark Annealing Buffer and PyroMark Wash Buffer (QIAGEN, Hilden, Germany). As biotin binding protein, Streptavidin Sepharose High Performance (Cytiva, Freiburg im Breisgau, Germany) was used. All primers were designed using the Pyromark Assay Design Software (QIAGEN, Hilden, Germany). The primer sequences are shown in Supplementary Table S1. The sequences of interest were selected by prior analysis of TCGA data by the following conditions: high difference in DNA methylation between tumor and normal tissue and correlation of DNA methylation status and mRNA expression. 

### 2.6. Statistical Analysis

Statistical analysis was performed using GraphPad Prism 8.0.2 Software (GraphPad Software, San Diego, CA, USA). Each data set was tested for normality distribution using the Shapiro–Wilk test. To compare three normality distributed groups, one-way ANOVA test was used. For non-normality distributed data sets of two groups, the Mann–Whitney U test and, for three groups, the Kruskal–Wallis test was used. In case of multiple group comparisons, Tukey´s multiple comparison test and Dunn´s multiple comparison test were performed as post hoc tests. Due to incomplete data for metastases, methylation data of the analyzed CpG sites were considered as unmatched. Two-sided *p*-values ≤ 0.05 were assumed to be statistically significant and all error bars were illustrated as standard error of mean (SEM). To calculate statistical correlations between DNA methylation and mRNA expression, Spearman’s rank correlation coefficient was used. For evaluation of survival data, the Kaplan–Meier method with log-rank test was performed.

## 3. Results

### 3.1. Identification of Potentially Clinically Relevant CpG Islands in the SFRP1 Promoter in Pancreatic Cancer

**Figure 1:**
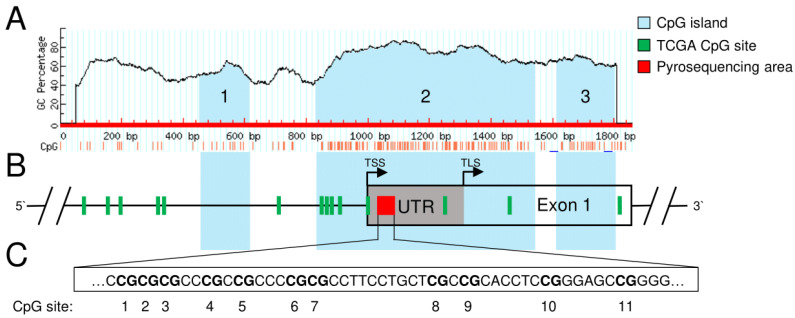
Localization of identified CpG islands and TCGA CpG sites in the region of interest of the genomic *SFRP1* sequence (exon 1 and 1000 bp upstream). (**A**) Three identified CpG islands by default criteria of CpG island size > 100 bp, CG percentage > 50% and observed to expected CpG ratio > 60%. CpG islands are labelled in blue and numbered upstream to downstream (1–3) in the schematic *SFRP1* gene map. (**B**) Additionally, positions of 14 CpG sites from the TCGA pancreatic cancer data set are labelled in green in the region of interest. Black line indicates promoter region upstream of transcription start site (TSS) as well as intronic sequence downstream of exon 1. The box indicates exon 1, including its grey marked 5′UTR (TLS denotes the translation start site). Note that seven CpG sites are covered by CGI2 (island 2 CpGs). The red box represents the area for methylation analysis, which is located in the 5′UTR of the first exon. (**C**) The area for methylation analysis (pyrosequencing assay) contains 11 selected CpG sites (numbered upstream to downstream, 1–11).

Performing in silico analysis of the genomic *SFRP1* sequence, we were able to identify three different CpG islands (abbreviated as CGI1, 2 and 3) in the range of exon 1 and 1000 bp upstream of the transcription start site (TSS) (Figure 1A). CGI1 (nucleotides 454–613) is 160 bp in size and located upstream of the TSS of exon 1. CGI2 (nucleotides 831–1541) contains 711 bp and overlaps the TSS and more than half of exon 1, including the noncoding 5′UTR of it. CGI3 (nucleotides 1612–1799) is 188 bp in size and located at the end of exon 1. Within the TCGA data set of 184 primary pancreatic tumor patients, we found 14 different CpG sites in the region of interest. However, only seven of these (cg10406295, cg17816908, cg21517947, cg01495122, cg24319902, cg22418909, cg15839448) are located in the sequence of one of the identified CpG islands (CGI2, Figure 1B). Therefore, we focused on these seven CpG sites in CGI2 for further analysis. All DNA methylation levels were determined as beta values, with a value of 1 corresponding to complete DNA methylation and a value of 0 corresponding to complete lack of DNA methylation. To determine the methylation status of specific DNA regions, the beta values of the affected CpGs were combined as mean beta value.

**Figure 2:**
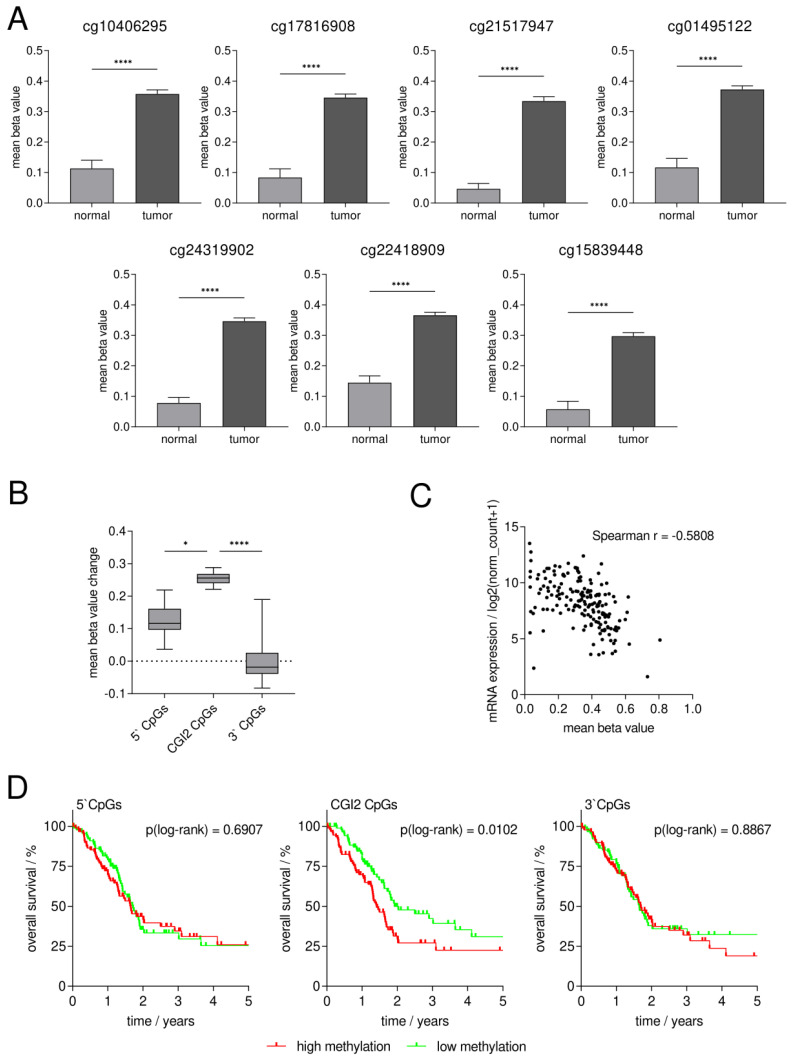
In silico analysis of *SFRP1* promoter methylation in primary pancreatic cancer shows significantly higher DNA methylation at specific CpG sites (CGI2) in tumor vs. normal tissue with potential clinical impact. (**A**) Mean methylation status of all specific CpG sites is significantly higher in tumor (*n* = 184) vs. normal tissue (*n* = 10) (Mann–Whitney *U* test, **** *p* < 0.0001). (**B**) Mean methylation change of CGI2 CpGs is significantly higher than that of 5’CpGs (Dunn’s test, * *p* < 0.05) and 3’CpGs (Dunn’s test, **** *p* < 0.0001). (**C**) Inverse correlation of mean CGI2 CpGs methylation status and *SFRP1* mRNA expression in primary pancreatic tumors (*n* = 178, Spearman r = −0.582). (**D**) Kaplan–Meier analysis in dependence of pancreatic cancer patients’ overall survival and their *SFRP1* DNA methylation status dichotomized by the median in high (red) and low (green) methylation. Left and right: no significant difference of overall survival between high vs. low methylation status of 5’CpGs (median beta value = 0.31, log-rank test, ns *p* = 0.6907) and 3’CpGs (median beta value = 0.56, log-rank test, ns *p* = 0.8867). Middle: significantly longer overall survival with low vs. high methylation status of CGI2 CpGs (median beta value = 0.37, log-rank test, * *p* = 0.0102).

For all seven CGI2 CpG sites, we detected a significantly higher mean methylation status in tumor (0.30 to 0.37, *n* = 184) compared to normal tissue (0.05 to 0.14, *n* = 10) (Mann–Whitney *U* test for all CpGs **** *p* < 0.0001, Figure 2A). Furthermore, we found that the mean methylation change from pancreatic normal to tumor tissue of the CGI2 CpGs (0.22 to 0.29) is significantly higher than the mean methylation change of the 5’CpGs upstream of CGI2 (13 CpGs with 0.04 to 0.22) and the 3’CpGs downstream of CGI2 (15 CpGs with −0.08 to 0.19), pointing out the crucial role of CGI2 in *SFRP1* promoter methylation in pancreatic cancer (Figure 2B) (overview of all TCGA CpG sites in Supplementary Table S3). Additionally, we found an inverse correlation (*n* = 178; Spearman r = −0.5808, 95CI: −0.6730 to −0.4709; **** *p* < 0.0001) between the mean CGI2 methylation status and the mRNA expression of *SFRP1* (Figure 2C). 

We performed Kaplan–Meier analysis in dependence of the overall survival of the pancreatic cancer patients and their *SFRP1* methylation status dichotomized by the median in low and high methylation. In dependence of the CGI2 CpGs in the *SFRP1* promoter sequence, we found that patients with low methylation status showed significantly longer overall survival compared to those with high CGI2 methylation status (* *p* = 0.0102). The median overall survival of the high *SFRP1* CGI2 methylation cohort (*n* = 92) is 517 days while the one of the low methylation cohort (*n* = 92) is 702 days. In dependence of the 5’CpGs or the 3’CpGs, we found no significant differences between the low methylation cohort (each *n* = 92) with a median overall survival of 627 days and 603 days compared to the high methylation cohort (each *n* = 92) with a median overall survival of 603 days (ns *p* = 0.6907) and 619 days (ns *p* = 0.9735) (Figure 2D).

### 3.2. Confirmation of DNA Hypermethylation of a Core CpG Island in SFRP1 Analyzing an Independent Collective of Pancreatic Cancers

**Figure 3:**
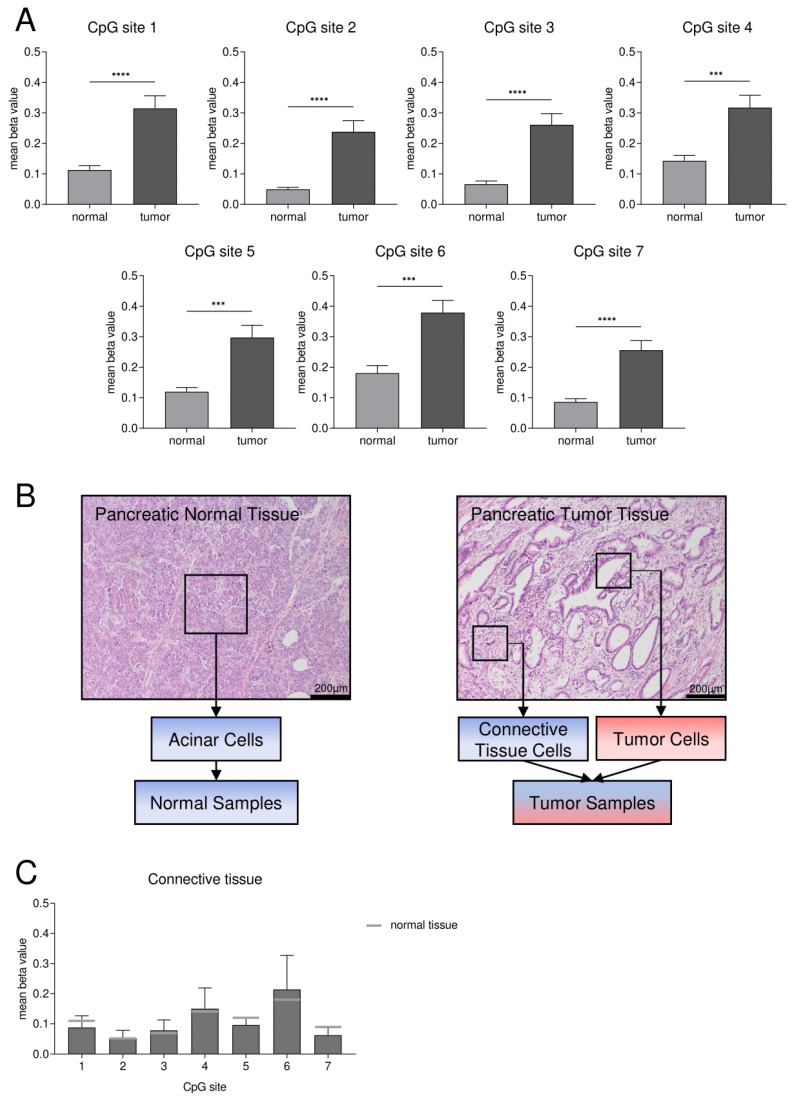
Abundant *SFRP1* CGI2 DNA methylation in pancreatic cancer patients compared to normal acinar tissue. (**A**) Mean methylation status of seven specific CpG sites (CpG site 1–7, Figure 1C) is significantly higher in pancreatic ductal adenocarcinoma (tumor) (*n* = 28) vs. normal tissue (*n* = 28) (Tukey’s test for CpGs 1, 2, 4, 5, 7, Dunn’s test for CpGs 3, 6, *** *p* < 0.001, **** *p* < 0.0001). Since the three groups of normal, tumor and additionally metastases tissues were compared, statistical analysis was performed as multiple comparison. (**B**) HE-stained microscopic images of pancreatic normal acinar tissue and pancreatic ductal adenocarcinoma with connective tissue fraction. The boxes indicate the macrodissected cells used for DNA isolation (blue = low *SFRP1* methylation, red = high *SFRP1* methylation). (**C**) Mean DNA methylation status of the CpG sites 1–7 for isolated connective tissue vs. normal tissue (grey) is comparable.

To validate our in silico results, we designed a pyrosequencing assay according to the sequence of the identified core CpG island (CGI2) with eleven CpG sites (Figure 1C). Subsequently, we analyzed the DNA methylation status of 28 cases of pancreatic ductal adenocarcinoma tissues compared to the corresponding pancreatic normal acinar tissues that underwent surgery at the University Hospital RWTH Aachen. We performed the methylation analysis on isolated DNA of FFPE specimens and found that CpG sites 1–7 showed the best technical reliability. We found a significantly higher mean methylation status in tumor tissues (*n* = 28) with 0.24 to 0.38 than in normal tissues (*n* = 28) with 0.05 to 0.18 (Tukey’s test for CpGs 1, 2, 4, 5, 7, Dunn’s test for CpGs 3, 6, *** *p* < 0.001, **** *p* < 0.0001; choice of statistical test based on detected normality distribution) (Figure 3A). Additionally, we investigated the mean DNA methylation status of seven corresponding pancreatic metastases and found with 0.20 to 0.29 a significantly higher methylation status in metastases (*n* = 7) compared to normal tissue (*n* = 28) for the CpG sites 1, 2, 5 and 7 (Tukey’s test, * *p* < 0.05, ** *p* < 0.01), while the mean DNA methylation status of metastases vs. tumor was in a comparable range.

Tissues of pancreatic adenocarcinomas consist not only of tumor cells but also of connective tissue cells [28]. As DNA was isolated by macrodissection, there might be a potential impact of impurities of connective tissue cells on the DNA methylation analysis (Figure 3B). Therefore, we investigated the DNA methylation status of macrodissected connective tissues of five pancreatic adenocarcinoma samples as well. We found that the mean methylation status for the CpG sites 1–7 ranges from 0.05 to 0.21 and that the methylation pattern is comparable to the one of pancreatic normal acinar cells (Figure 2C). 

**Figure 4:**
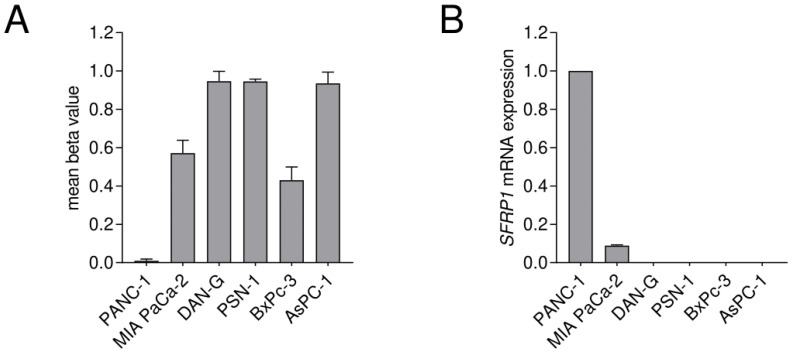
Comparison of CGI2 methylation status and *SFRP1* mRNA expression in pancreatic cancer cell lines. DNA methylation of *SFRP1* promoter core CpG island 2 was measured. (**A**) Mean *SFRP1* CGI2 methylation status shows abundant DNA methylation for the cell lines MIA PaCa-2, DAN-G, PSN-1, BxPc-3 and AsPC-1, while PANC-1 shows no methylation. (**B**) Relative *SFRP1* mRNA expression shows negligible expression for the cell lines DAN-G, PSN-1, BxPc-3, AsPC-1, while PANC-1 and MIA PaCa-2 show detectable expression (*n* = 3 for each cell line). The determination of the relative *SFRP1* mRNA expression is based on triplicates for each cell line and is normalized to PANC-1 as the highest expressing cell line.

Finally, the CGI2 methylation status was analyzed in a set of pancreatic cancer cell lines, where impurities of connective tissue cells can be excluded, as exclusively pure cancer cells are present. The DNA methylation status of the eleven selected CpG sites of CGI2 was investigated (Figure 1C). We found a high mean DNA methylation status for the cell lines DAN-G, PSN-1 and AsPC-1 with 0.94 to 0.95 and a medium status for the cell lines MIA PaCA-2 and BxPc-3 with 0.57 and 0.43, respectively. Interestingly, we found that the PANC-1 cell line, commonly used in pancreatic cancer research, does not show *SFRP1* promoter methylation at CGI2 (Figure 4A). Additionally, we analyzed the *SFRP1* mRNA expression of all cell lines and detected negligible *SFRP1* mRNA expression in DAN-G, PSN-1, AsPC-1 and BxPc-3 cells, while we measured relatively abundant *SFRP1* mRNA expression in PANC-1 cells and compared to that relatively low *SFRP1* mRNA expression in MIA PaCa-2 cells (Figure 4B). We observed an inverse trend between mean CpG island 2 methylation status and *SFRP1* mRNA expression, but the p-value was not significant in this small cohort of pancreatic cancer cell lines (*n* = 6, Spearman r = −0.7714, ns *p* = 0.1028).

## 4. Discussion

Until recently, the treatment of pancreatic cancer has only been the domain of surgical intervention. Nevertheless, the clinical importance of neoadjuvant and adjuvant chemotherapy continues to grow as new therapeutic concepts are explored and personalized treatment regimens become more widely applied [29]. In the context of personalized therapies based on genetic alterations, only some mutations are clinically relevant so far. For example, pancreatic cancer patients appear to benefit especially from platinum-based chemotherapy when a BRCA-1/2 germline mutation is present [30,31]. Patients with a verified mismatch repair deficiency appear to respond better to immune checkpoint inhibitors, which otherwise would not be a treatment option [30,32]. Besides these genomic sequence alterations, epigenetic alterations are common and seem to be crucial for pancreatic cancer development and progression as well. These epigenetic changes occur in multiple ways such as histone modifications, altered expression of miRNAs and altered DNA methylation [33,34,35]. In the case of altered DNA methylation, the pro-oncogenic effect occurs typically through the inactivation of genes by promoter DNA hypermethylation [36]. Therefore, DNA methylation analysis in pancreatic cancer may become a predictive biomarker for therapy response like DNA methylation is in the case of glioblastomas, a common cancer of the brain. Here, based on the DNA methylation status of the *O^6^-methylguanine-DNA methyltransferase* (*MGMT*) gene promoter region, the clinical response of glioblastomas to temozolomid chemotherapy clearly differs and, therefore, affects the choice of chemotherapy [37]. Malley et al. were able to identify two regions, so-called DMR1 and DMR2, inside the *MGMT* promoter region, which are crucial for gene silencing through promoter DNA hypermethylation [38]. So far, the class 2 tumor suppressor gene *SFRP1* has been described as a frequently methylated gene in pancreatic cancer, either directly in cancerous tissue [23] or in blood-based plasma-derived cell-free DNA [27]. The blood-based DNA methylation analysis of Stubbe et al. also suggested *SFRP1* as a promising independent predictive marker for survival in end stage pancreatic cancer patients treated with gemcitabine [27]. However, a detailed molecular analysis of the *SFRP1* promoter region hypermethylated in pancreatic cancer has not been performed yet. Therefore, this study was designed to identify crucial regions of *SFRP1* CpG site hypermethylation and further evaluate their potential clinical impact in pancreatic cancer. 

In our study, we were able to identify three different regions inside the *SFRP1* promoter region of interest that could act as a CpG island and, therefore, regulate SFRP1 expression. Out of those, we focused on CpG island 2 (CGI2) due to the short length of CpG island 1 and 3 (<200 bp). Illingworth et al. provided an overview of different sources that assumes a certain CpG sequence length as a CpG island. Most commonly, at least 200 bp or more were recommended, even though some sources use user-defined shorter lengths [39]. By increasing the minimal sequence length, identification of actual CpG islands is more precise due to the extraction of short, interspersed elements, which can comprise relatively high cytosine and guanine rates but do not act as a CpG island [40]. Additionally, CGI2 was selected due to its overlap of the transcription start site and the upstream part of the first exon. Especially in these regions, DNA methylation is more likely linked to transcriptional silencing [41]. In pancreatic cancer, this is reflected by our finding of a strong inverse correlation between DNA methylation status of CGI2 and *SFRP1* mRNA expression. In addition, we found strong DNA methylation changes in CGI2 compared to the surrounding CpGs underlining the potential impact of this identified core CpG island in regulating *SFRP1* expression in pancreatic cancer. The SFRP1 promoter region, which we focused on for DNA methylation analysis in pancreatic cancer, has only been analyzed in a study for renal cell cancer [42]. The analyzed CpG sites of this study are located in the region of our identified core CpG island, but they are upstream of the transcription start site. The authors demonstrated enhanced DNA methylation in different tumor development stages of renal cell cancer. Additionally, they proposed the DNA methylation status of this analyzed CpG island within the *SFRP1* gene as a predictor for recurrence-free survival [42]. In pancreatic cancer, our in silico analysis for *SFRP1* revealed favorable overall survival of those patients exhibiting low DNA methylation in the core CpG island (CGI2), highlighting the potential clinical impact of *SFRP1* CpG site-dependent promoter methylation as a prognostic or predictive biomarker. 

To validate our in silico findings, we performed pyrosequencing on bisulfite-treated DNA samples from pancreatic cancer tissues and cell lines based on our identified core CpG island. In the pancreatic cancer tissue, we demonstrated a significantly higher *SFRP1* promoter DNA methylation in CGI2 than in normal acinar tissue. However, the mean beta values of the cancer tissues are below the defined cut-off values (0.5–0.7) for stable gene expression silencing by DNA hypermethylation [43,44]. Pancreatic cancer is known for its strong desmoplastic reaction, leading to a high proportion of connective tissue in the primary tumor samples [28]. Therefore, and due to our macroscopic dissection of FFPE tissues, a proportion of connective tissue cells cannot be completely excluded for DNA isolation even though we demonstrated a high ratio of cancer to connective tissue cells. Previous gene expression studies of stromal tissue from pancreatic cancer found *SFRP1* to be downregulated in pancreatic cancer-associated connective tissue and assumed abundant DNA methylation as a possible mechanism but did not actually investigate *SFRP1* promoter methylation [45]. However, we could not verify their assumption, as we detected similar low DNA methylation levels for cancer-associated connective tissue cells and normal pancreatic acinar cells. Thus, the proportion of connective tissue cells as a possible contamination in the isolation of pancreatic cancer cell DNA might lead to falsely low DNA methylation levels below the typically cut-off values. Our in vitro analysis of the identified core CpG island revealed three pancreatic cancer cell lines with a CGI2 methylation status greater than 0.9, indicating complete DNA methylation in this region. In comparison to pancreatic cancer tissue, the isolated cell line DNA exclusively consists of pancreatic cancer cells and does not contain any non-tumor cells. Hence, DNA methylation of *SFRP1* in pancreatic cancer cells seems to be a prominent epigenetic modification. Thus, connective tissue cell contamination in isolated DNA of pancreatic cancer tissue might be a reason for DNA methylation levels below typical cut-off values. Interestingly in a single pancreatic cancer cell line (PANC-1), we found nearly no CGI2 methylation and concordantly abundant mRNA expression of *SFRP1*, which contrasts previous MSP studies [23]. In summary, our study shows that the identified core CpG island CGI2 of *SFRP1* might be a promising tool for the development of clinical assays in pancreatic cancer prognosis and treatment response prediction.

Since pancreatic cancer is associated with low survival rates [2], there are already many studies trying to develop clinical prognostic tests. For example, Henriksen et al. have shown that cell-free DNA hypermethylation of several genes, among these *SFRP1*, was associated with stage-dependent poor overall survival [46]. More precisely, Stubbe et al. were able to show a significant association between hypermethylated *SFRP1* in cell-free DNA and poor responsiveness to gemcitabine therapy in stage IV pancreatic cancer [27]. Most commonly, the *SFRP1* methylation analyses are performed by using MSP on cell-free DNA derived from blood samples. Even though this liquid biopsy method can easily be applied after a blood withdrawal, its analytical sensitivity is low, as cell-free DNA (cfDNA) in the blood is mainly composed of DNA derived from normal cells, and only a tiny fraction, called ctDNA, is derived from tumor cells [47]. Since most pancreatic tumors are surgically resected, FFPE tissues (available in pathology institutes) could also be used after a few preparation steps. It may be advantageous to perform *SFRP1* promoter methylation analysis directly on DNA derived from cancerous tissue to increase detection sensitivity. It should be added that our pyrosequencing assay was designed to work well on fragmented DNA from FFPE tissues. An additional advantage of the pyrosequencing method is that the DNA methylation status of a target gene such as *SFRP1* can be resolved at the level of single CpG sites in a stretch of about 100 base pairs [37]. These precise and detailed DNA methylation data may help to better stratify pancreatic cancer samples in hypermethylated or not, which may be more difficult using MSP [37]. 

Our study examined the genomic *SFRP1* sequence and its potential regions for de novo DNA methylation in pancreatic cancer with key effects on SFRP1 expression silencing. In silico, we identified a core CpG island (CGI2) covering the transcription start site and the first half of exon 1. DNA methylation of this CpG island appears to have a decisive effect on the loss of *SFRP1* mRNA and protein expression, leading to a significant influence on patients’ overall survival. In vitro, we confirmed the altered DNA methylation of the core CpG island based on pancreatic cancer patient tissues and pancreatic cancer cell lines, proposing a new pyrosequencing assay. This assay could be used for future FFPE or blood-based DNA methylation studies, which, for example, could help to determine the responsiveness to chemotherapeutic treatment in certain patient groups. 

## 5. Conclusions

Treatment decisions based on the molecular alterations of cancer cells are the future of modern cancer therapy. While it is already standard practice for several cancer entities to identify driver mutations and apply therapeutic regimens accordingly, both predictive markers and personalized therapy options are still limited in pancreatic cancer. *SFRP1* promoter hypermethylation represents a potential biomarker that may help to stratify pancreatic cancer patients for chemotherapy response. We hope that the provided information will support future assay development to bring *SFRP1* methylation analysis to a clinical application in pancreatic cancer.

## Data Availability

The data supporting the study findings are available from the authors upon reasonable request.

## Supplementary Materials

The following are available at https://www.mdpi.com/article/10.3390/cancers15030683/s1, Table S1: Primer sequences for RT-PCR and pyrosequencing; Table S2: Clinico-pathological parameters of the FFPE patient collective; Table S3: List of CpG sites per region in *SFRP1* sequence.
