# Supplementary material for: Identification and Validation of Potentially Clinically Relevant CpG Regions within the Class 2 Tumor Suppressor Gene SFRP1 in Pancreatic Cancer
Source: Cancers (Basel). 2023 Jan 22;15(3):683. doi: 10.3390/cancers15030683 (PMC9913221; doi:10.3390/cancers15030683)
Supplement: Supplementary file 1 [file cancers-15-00683-s001.zip › cancers-2142083-supplementary.pdf]

1 Supplementary

Table S1. Primer sequences for RT-PCR and pyrosequencing.

Method	Gene	Direction	Sequence
rtPCR	SFRP1	forward	5'-AGATGCTTAAGTGTGACAAGTTCC-3'
		reverse	5'-TCAGATTTCAACTCGTTGTCACAG-3'
	ACTB	forward	5'-TGA CGT GGA CAT CCG CAA AG-3'
		reverse	5'-CTGGAAGGTGGACAGCGAGG-3'
Pyrosequencing	SFRP1	forward	5'-AGGGTTYGGTYGTAGGAGTTT-3'
		reverse	5'-ATCCCCCCRACCAATAAC-3'
		sequencing	5'-TGTAGTTTTYGGAGTTAGTG-3'

Table S2. Clinico-pathological parameters of the FFPE patient collective with 28 cases of pancreatic ductal adenocarcinomas.

Category	Parameter	n
Age at diagnosis	<50	1
	50-59	3
	60-69	10
	70-79	12
	80-89	2
Gender	Male	15
	Female	13
Histological tumor grade		
G	1	1

	2	12
	3	15
Histological tumor stage		
pT	1	0
	2	4
	3	23
	4	1
pN	0	1
	1	27
pL	0	18
	1	9
pV	0	23
	1	5
Pn	0	2
	1	25

Table S3. List of CpG sites per region in *SFRP1* sequence based on TCGA for *in silico* analysis (Infinium HumanMethylation450 BeadChip data).

Region	CpG sites
5´CpGs	cg07122178, cg23359714, cg00930833, cg09410389, cg03575666, cg01074584, cg07935886, cg14824386, cg06166767, cg00000321, cg03133371, cg14904908, cg04255616
Island 2 CpGs	cg10406295, cg17816908, cg21517947, cg01495122, cg24319902, cg22418909, cg15839448
3`CpGs	cg13398291, cg02388150, cg14548509, cg16662821, cg21846232, cg06777844, cg13154925, ch,8,969355F, cg16498741, cg17486234, cg02154585, cg23331238, cg07296835, cg25927227, cg16667459
